# In-Vitro Cytotoxicity, Apoptotic Property, and Gene Expression Changes Induced by Naringenin-7-O-Glucoside in Triple-Negative Breast Cancer

**DOI:** 10.7759/cureus.58634

**Published:** 2024-04-20

**Authors:** Akhila R James, Sakthidasan Jayaprakash, Lakshmi M Sundeep

**Affiliations:** 1 Biotechnology, Hindustan Institute of Technology and Science, Chennai, IND

**Keywords:** selective cytotoxicity, anticancer potential, egfr downregulation, naringenin-7-o-glucoside, triple-negative breast cancer

## Abstract

Introduction: Cancer is one of the most significant health challenges demanding the expansion of effectual therapeutic methods. Triple-negative breast cancer (TNBC) is a form of aggressive cancer with inadequate therapeutic options which lacks the expression of certain hormones.

Materials and methods: The present study investigates the potential of naringenin-7-O-glucoside, a flavanone glycoside extracted from *Holarrhena antidysenterica *as an anticancer agent against TNBC cell lines. In-vitro analysis to evaluate cytotoxicity, apoptotic-inducing properties and effect on gene expression was conducted.

Results: MTT (3-(4,5-dimethylthiazol-2-yl)-2,5-diphenyltetrazolium bromide) assay studied the IC-50 of naringenin-7-O-glucoside to be 233.56 µg/µL, revealing the dose-dependent cytotoxicity with minimal effect on Vero cells. Extensive DNA fragmentation confirmed the apoptotic property. Furthermore, a significant downregulation of the epidermal growth factor receptor (EGFR) was noted in treated cells when equated to the control specimen of the sample.

Conclusion: Therefore, naringenin-7-O-glucoside can be a potential targeted therapeutic agent.

## Introduction

Cancer is the primary health issue with a significant global impact in both developed and developing nations. A higher incidence rate has impacted the therapy and prognosis [1].

Breast cancer (BC) accounts for the primary cause of cancer mortality in females when compared to men. Based on gene expression and genetic mutations expressed in cancer cells, BC can be categorised into various molecular subtypes [2]. Hormone receptor-positive, triple-negative breast cancer (TNBC), basal-like, luminal A, luminal B, and claudin-low are some subtypes of BC [3]. TNBC is among the most difficult type of BC distinguished by the absence of estrogen receptor, progesterone receptor and human epidermal growth factor receptor 2 expression. As a result, TNBC does not respond to hormonal therapies or targeted therapies [4]. TNBC tends to transpire more frequently in younger women, women with a BRCA1 gene mutation, and African American women. The subtype accounts for roughly 10-20% of total BC occurrences [5]. Treatment for TNBC typically involves chemotherapy such as anthracyclines and taxanes, surgery, and radiotherapy. But currently used chemotherapeutic drugs like doxorubicin, paclitaxel, eribulin and radiation cause a lot of side effects affecting the overall life and health standard of the patient [6].

The primarily available treatment options include hormones, targeting agents such as PI3K/AKT/mTOR inhibitors and notch inhibitors, immunotherapies, DNA interacting agents and anti-tubulin agents [7]. Synthetichemotherapeutic agents, commonly used in treatment, carry various side effects including nausea, fatigue, mouth sores, diarrhoea, anaemia, and neuropathy. Additionally, it can also lead to severe complications such as anaphylaxis, cardiac issues, liver and kidney damage, and life-threatening infections [8-11]. Therefore, using phytochemicals as anticancer agents will fight cancer without causing any severe side effects.

Phytochemicals are plant derivatives and are assuring potentials to enhance treatment efficacy and bring down adverse side effects. Plants have been utilized for the remedy of numerous diseases from time immemorial. Traditional Indian Medicine, Ayurveda and Traditional Chinese Medicine persists as the oldest yet existing traditions, dating back to 4500 BC. In ancient times, the identification and selection of suitable plants, drug preparation methods and their respective medicinal use were transmitted verbally across consecutive generations. The folklore documented comprehensive data related to drugs and their applications for numerous medicinal conditions. The drugs were procured in various forms, including teas, decoctions, tinctures, powders, poultices, and other preparations.

Many phytochemicals like curcumin and quercetin have been found to have corresponding and overlapping methods to bring down the oncogenic process by attacking free radicals, reducing survival and growth of malignancies, while also limiting invasiveness and angiogenesis of tumours. The phytochemicals are found to reverse the epigenetic alterations or halt the downstream process without severe side effects [12,13].

## Materials and methods

Cell line culture

The Vero, MDA-MB-231 and MCF-7 cell lines were procured from the National Centre for Cell Science (NCCS), Pune, India and kept sterile and were maintained in accordance with accepted techniques in a CO_2_ incubator at 390°C with 5% carbon dioxide. Culturing of the cells was performed to meet the experimental criteria [14].

Cytotoxicity assay

The cell death assay calculates the degree of cellular impairment brought on by a compound on the cultured cell lines. MCF-7 and MDA-MB-231 cell lines derived from BC and Vero cell lines were retrieved from NCCS Pune for the conduction of the study [15,16].

A fast colourimetric assay utilising MTT (3-(4,5-dimethylthiazol-2-yl)-2,5-diphenyltetrazolium bromide) was performed to evaluate the compound's cytotoxicity on the cell lines, and the findings were compared to untreated controls. The cells from cell lines were cultured in 96-well plates at a concentration of 10,000 cells per well, comprising 100 mL of medium supplemented with 5% fetal bovine serum (FBS) for the screening experiments in each well. The compound naringenin-7-O-glucoside was then introduced into the cells after a 48-hour incubation period at 37°C, 95% air, 5% CO_2_, and 100% relative humidity. Following 48 hours, naringenin-7-O-glucoside was introduced and incubated for 48 hours by following the standard procedure. For accuracy, three replicates of both the treated and control groups were maintained.

After 48 hours, to each well, 50 μL of MTT solution (5 mg/mL) prepared in triple-distilled water was added, followed by four-hour incubation at 37°C. Upon removal of the MTT solution, the formazan crystals were dissolved in 100 μL of dimethyl sulfoxide (DMSO). Subsequently, by means of a microplate reader, the absorbance was measured (570 nm). The provided formula was used to derive the cell inhibition [17]:

$$Percentage\;cell\;inhibition= 100-{(At-Ab)/ (Ac-Ab)}*100$$

where At is the absorbance value of the test compound; Ab is the absorbance value of the blank; Ac is the absorbance value of control; absorbance of the blank was considered as 0.

DNA fragmentation assay

Control and treated cells were trypsinized and rinsed with 1X phosphate-buffered saline (PBS). A total of 200µl of cell lysis buffer and 20µl proteinase K were supplemented to the cell pellet followed by mixing by means of vortexing. The product was further incubated at 65°C for one hour. DNA was isolated with 400µl of phenol/chloroform/isoamyl alcohol mixture (25:24:1, v/v/v) and then centrifuged at 12,000 rpm for 15 mins. The upper aqueous layer containing DNA was moved into another microfuge tube containing 500µl of ice-cold isopropanol and 50µl of sodium acetate. DNA was pelleted down by centrifugation at 12,000 rpm for 15 mins. The DNA pellet was then rinsed with 70% ethanol at 13,000 rpm for five minutes [18].

The air-dried pellet was dissolved in 20µl of 1X TE (Tris EDTA) buffer. DNA fragmentation was visualized by agarose (1.5%) gel electrophoresis.

Gene expression analysis

The gene expression analysis of the compound against the EGFR was determined using RNA isolation, cDNA techniques and reverse transcription-polymerase chain reaction (RT-PCR) [19].

Total RNA isolation

Total RNA from cells was isolated as described by Chomczynski and Sacchi method [20]. The entire procedure was conducted in an environment which was ribonuclease (RNase)-free. Cells were directly added to a monophasic acidic phenol-guanidinium thiocyanate-based Affypure TriReagent (Affyclone Laboratories Pvt Ltd., Chennai, India) and mixed thoroughly using a diethylpyrocarbonate (DEPC)-treated hand homogenizer. The sample was then lysed by repeated pipetting and kept standing for five minutes at room temperature, succeeded by the supplementation of 200μL chloroform for phase separation. Upon the addition of chloroform, the mixture separated into three phases, with RNA located in the aqueous phase. The RNA-containing aqueous layer was carefully transferred to a new sterile microfuge tube treated with DEPC, followed by the addition of 250μL of ice-cold isopropanol. After vigorous vortexing, the mixture was allowed to stand for 15 minutes at 35°C. Subsequently, the solution was centrifuged at 12,000g for 15 minutes at 4°C. After centrifugation, the supernatant was discarded, leaving behind the RNA pellet. The pellet was rinsed thoroughly, and subjected to centrifugation at 14,000g for 10 minutes at 4°C. The ethanol wash was repeated, followed by a five-minute centrifugation. The RNA pellet was air-dried and then reconstituted in DEPC-treated water by heating for 20 minutes. Subsequently, the RNA solution was preserved at -80ºC for later examination. The RNA's purity was assessed using a UV spectrophotometer.

Conversion of cDNA and RT-PCR

The synthesis of cDNA commenced with a reverse transcription process facilitated by the GeNei™ cDNA conversion kit (Genei Laboratories Pvt Ltd., Bangalore, India). Firstly, a reaction mixture comprising 0.5-1.0μg of total RNA and 1.5μL of primer underwent incubation for 10 minutes at 72°C, followed by immediate cooling. Subsequently, 5.0μL of a pre-mixed 10 mmol/L deoxynucleotide triphosphate (dNTP) solution, 3.0μL of 10× Moloney Murine Leukemia Virus reverse transcriptase (M-MLV RT) buffer, and 1.0μL of M-MLV RT were introduced to the mixture, which was then adjusted to a final volume of 50μL using sterile water which was free of RNase and deoxyribonuclease (DNase). The resulting cDNA was subsequently amplified through PCR employing the Takara PCR master mix (Takara Bio Inc., Kusatsu, Japan). Evaluation of the PCR products was performed using 2% agarose gel electrophoresis, and visualization was achieved under a UV transilluminator.

Experiments were performed in duplicates. Statistical analysis of the graphical data was expressed as the mean standard deviation. The p-value was analysed in comparison to the untreated using the Student t-test wherein p < 0.05 was considered as significant.

## Results

Cell cytotoxicity assay

**Figure 1:**
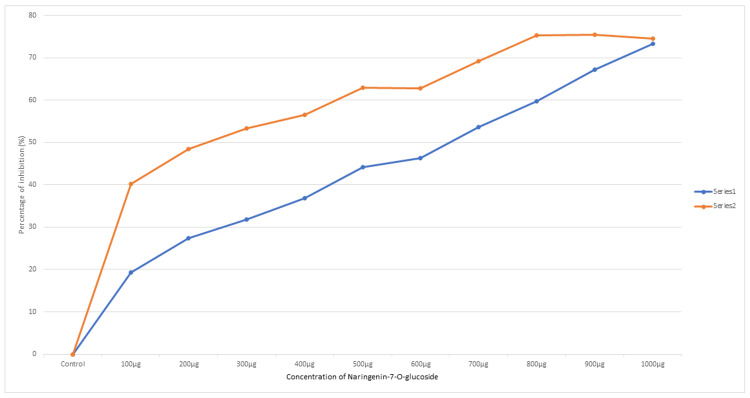
Dose-dependent inhibition curve of naringenin 7-O-glucoside on MDA-MB-231 and MCF-7 cell lines The optimum dose-dependent curve illustrates the percentage of inhibition of breast cancer cell lines against naringenin-7-O-glucoside. The dose-dependent curve shows how the percentages of inhibition change as the concentration of naringenin 7-O-glucoside increases from 0 (control) to 1000 µg. The X-axis represents the concentration of the compound and the Y-axis represents the percentage of inhibition on treatment. The control represents the untreated cells, serving as a baseline reference. Series 1 represents the MCF-7 cell line and series 2 represents the MDA-MB-231 cell line.

**Figure 2:**
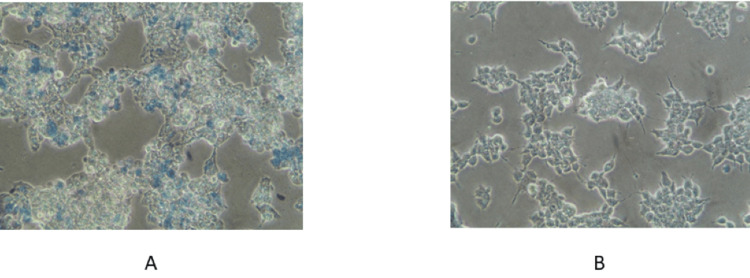
Naringenin-7-O-glucoside treated (A) MDA-MB-231 cell line and (B) MCF-7 cell line The image illustrates the effect of the compound against (A) MDA-MB-231 and (B) MCF-7 cell lines. Both cell lines show cell death at the IC-50, but extensive cell death is observed in the MDA-MB-231 cell line compared to the MCF-7 cell line. This observation suggests a greater susceptibility of MDA-MB-231 cells to the cytotoxic effects of naringenin-7-O-glucoside. The image was captured at a magnification of 20X and a scale of 5 microns.

The MCF-7 BC cell line, the MDA-MB-231 cell line, and the Vero cell line were used to test the cytotoxicity activity of naringenin 7-O-glucoside, and the IC-50 value was calculated. After 48 hours, the values were determined using a dose-response inhibition curve. On the TNBC cell line MDA-MB-231, the naringenin 7-O-glucoside exhibited an IC-50 value of 233.56 µg/µL, on the MCF-7 cell line, 698.44 µg/µL, and on the Vero cell lines, 1196.52 µg/µL (Table 1). Figure 1 depicts the exposure of two different cancer cell lines to naringenin 7-O-glucoside and the resulting dose-response curve. The impact of naringenin 7-O-glucoside on the MDA-MB-231 and MCF-7 cell lines is illustrated in Figures 2A-2B.

**Table 1:** IC50 values of naringenin-7-O-glucoside on MDA-MB-231, MCF-7 and Vero cell lines

Sample	IC_50_ (µg/µL)		
	MDA-MB-231	MCF-7	Vero cell line
Naringenin-7-O-glucoside	233.56	698.44	1196.52

DNA fragmentation analysis

**Figure 3:**
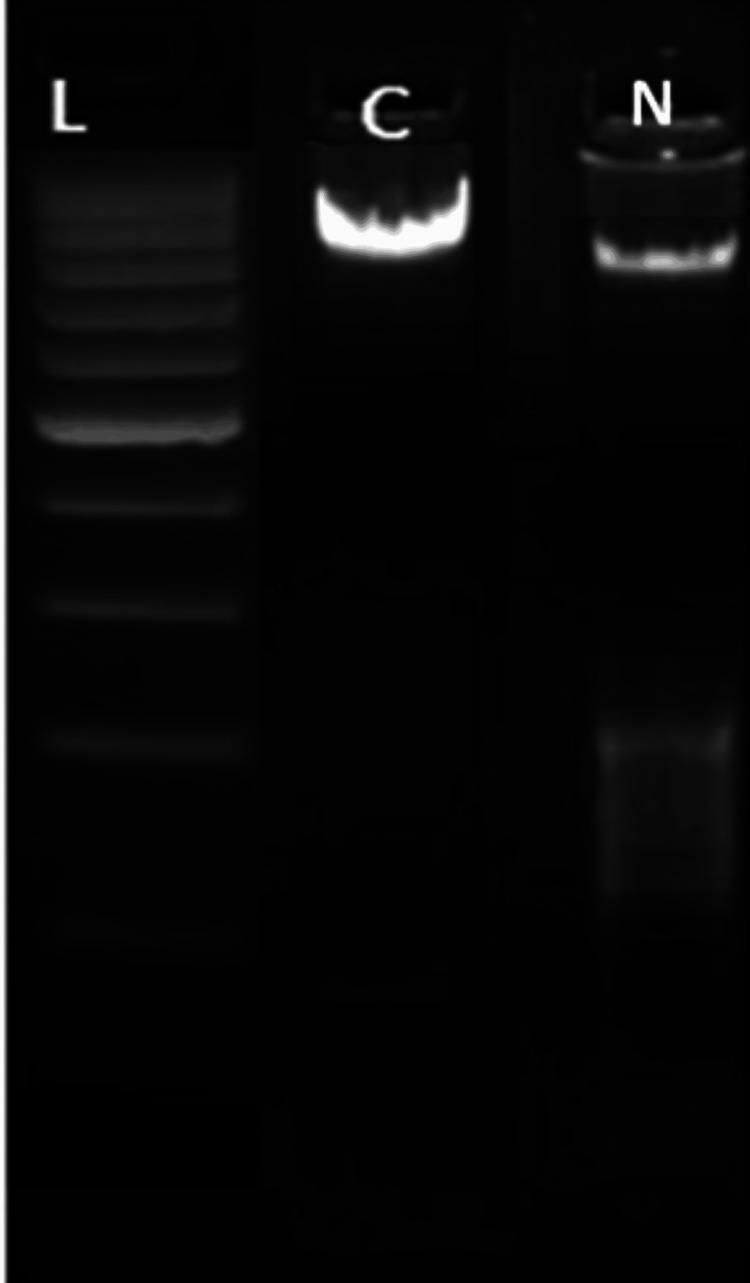
DNA fragmentation analysis using gel electrophoresis showing ladder, control and treated cell lines The figure shows the effect of naringenin-7-O-glucoside on cellular DNA integrity. The three lanes, namely L, C and N, represent the ladder lane having DNA fragments of known size for reference (100bp to 1000bp); the control lane consisting of untreated MDA-MB-231 cells providing baseline comparisons and cell lines treated with naringenin-7-O-glucoside respectively. The treated lane (N) shows fragmentation of DNA indicating apoptosis of cell lines when treated with the compound. The control lane (C) exhibits minimal fragmentation, consistent with intact DNA in untreated cells. The ladder lane (L) serves as a reference for fragment size determination.

DNA fragmentation analysis, a characteristic of DNA apoptosis, was studied on the MDA-MB-231 TNBC cell lines. The DNA treated and control was analysed using agarose gel electrophoresis. Both the dead and the live cells were taken for the study. As shown in Figure 3, fragmented DNA is seen at 800bp and a smear is observed between 100bp and 200 bp indicating extensive DNA fragmentation when treated with naringenin-7-O-glucosidein. The results suggest that naringenin-7-O-glucoside is a potent apoptotic inducer in MDA-MB-231 TNBC cell lines.

Gene expression analysis assay

RNA isolation, cDNA synthesis, and RT-PCR techniques were used for analyzing the gene expression of the compound against EGFR.

In the method, total RNA was isolated from cells as described by Chomczynski and Sacchi [14]. To initiate the procedure, cells were lysed with monophasic acidic phenol-guanidinium thiocyanate-based Affypure TriReagent. Chloroform was put in to separate the RNA-containing aqueous phase which was then precipitated with isopropanol. After centrifugation, the RNA pellet was rinsed with ethanol and dissolved in DEPC-treated water. The purity of isolated RNA was determined using a UV spectrophotometer. The absorbance was measured at A260 and A280 and the ratio was 1.9.

Isolated RNA samples were converted into cDNA using a cDNA conversion kit (GeNei™). Random hexamer primers and M-MLV RT were used to reverse transcribe total RNA (0.5-1.0μg). Takara PCR master mix then amplified resultant cDNA products. The PCR products were visualized on agarose gels under UV transillumination.

**Figure 4:**
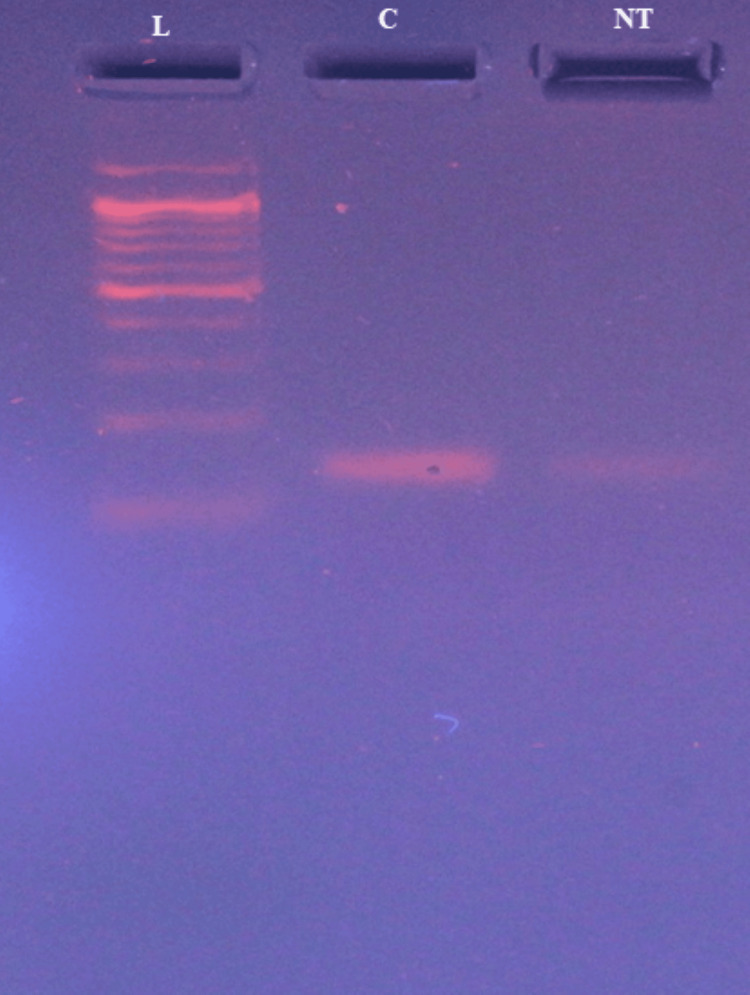
Gene expression analysis demonstrating downregulation of EGFR when treated with naringenin-7-O-glucoside The figure illustrates gene expression analysis showing the downregulation of EGFR in the MDA-MB-231 cell line following treatment with naringenin-7-O-glucoside. Three lanes are presented: L represents the ladder lane, containing DNA fragments of known sizes for reference; C denotes the control lane serving as a baseline comparison; and NT indicates the treated lane with naringenin-7-O-glucoside. Distinct bands observed at 157 bp in the treated lane signify the downregulation of EGFR expression in response to naringenin-7-O-glucoside treatment. This reduction in EGFR expression suggests a potential inhibitory effect of the compound on EGFR signalling pathways. In contrast, the control lane shows consistent EGFR expression levels, indicating no treatment-induced alterations in gene expression. The ladder lane (L) aids in fragment size determination for accurate analysis. EGFR: epidermal growth factor receptor

**Figure 5:**
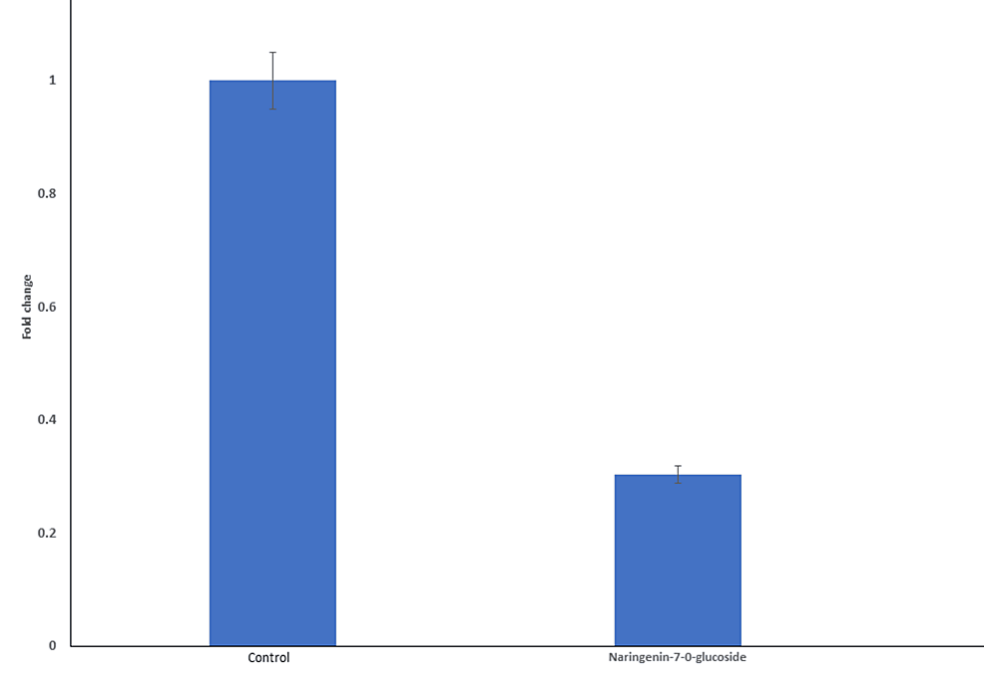
Densitometry analysis of EGFR expression levels The figure illustrates the densitometric analysis of EGFR expression in the MDA-MB-231 cell line when treated with naringenin-7-O-glucoside. Fold change values were calculated to quantify the relative expression levels compared to the control group, which has a fold change of 1. MDA-MB-231 cell lines treated with naringenin-7-O-glucoside show a fold change of 0.3, suggesting a substantial downregulation of EGFR expression in response to the treatment. The p-value for the observed difference in EGFR expression between the control and treated groups is <0.05, indicating statistical significance. EGFR: epidermal growth factor receptor

The expression level of EGFR in cells treated with the compound was compared to that of untreated control cells. From gel electrophoresis analysis, it emerged that there was reduced intensity of the EGFR band in treated cells relative to control cells at 157 bp (Figure 4). Densitometry analysis confirmed a significant reduction in EGFR expression levels in treated cells compared with controls (p<0.05) (Figure 5).

The observed downregulation of EGFR expression suggests a potential inhibitory effect of the compound on EGFR signalling pathways.

## Discussion

Cancer is a major lethal disease in the globe with various therapeutic options. Cancer is a complex disease with uncontrolled growth of cells leading to tumour microenvironment. The abnormal cellular growth can be a result of mutations in oncogenes which play a major role in cell regulation and cell cycle arrest. The mutations can be hereditary or epigenetic. The drugs aid in inhibiting cancer growth and progression. However, most of the synthetic chemicals are non-selective and can affect the healthy cells leading to various side effects. Therefore, investigating traditional medicine, practised for millennia, offers valuable insights into the potential of various plants against various ailments, including cancer. Some phytochemicals disrupt the uncontrolled cell cycle inducing apoptosis while some target the angiogenesis process eventually killing the tumour cells. Phytochemicals are also efficient in cribbing cancer by causing mitotic spindle destruction, cell cycle arrest at different phases, inhibition of cyclin-dependent kinases and many more. However, harnessing their full potential requires collaboration between traditional knowledge and modern scientific tools [21].

The research paper investigates the potential of naringenin-7-O-glucoside, a flavanone glycoside, as an anticancer agent against MDA-MB-231, a TNBC cell line. The results indicate significant antitumor efficacy, specifically targeting TNBC cells without affecting normal cells. The selective cytotoxicity confirms the compound as a viable therapeutic option. The study underscores the importance of naringenin-7-O-glucoside as a potential candidate for further exploration in cancer treatment in combating TNBC, a particularly aggressive form of BC.

Naringenin-7-O-glucoside exhibited significant dose-dependent cytotoxicity against the aggressive TNBC cell line MDA-MB-231, with minimal effect on the Vero-cell lines. Conventional chemotherapies often exhibit severe side effects due to cytotoxicity towards healthy cells making the selectivity of the compound crucial. Similar cytotoxic effects against TNBC cell lines have been reported in various studies. Cytotoxic studies on *Ardisia crispa* expressed an IC50 value of 139.57 ± 31.64 μg/mL in hydromethanolic extract and 347.44 ± 98.78 μg/mL in aqueous extract [22]. Similarly, chemotherapy results in various side effects in patients by affecting the healthy cells along with the cancer cells. First, cycle-induced consequences are nausea and vomiting (79.3%), fatigue (74.4%), reduced appetite (65.5%), hair loss (60.0%) and constipation (51.7%) [23]. Recurrence of disease with attained drug resistance is a major drawback in various chemotherapeutic drugs. The treatment may also lead to depression and anxiety [24].

Plant-based products and phytochemicals act as apoptotic agents and induce apoptosis [25]. The DNA fragmentation assay studies confirmed the cytotoxicity assay results by providing significant insights into the apoptotic-inducing potential of naringenin-7-O-glucoside against the TNBC cell line. The observed pattern of DNA fragmentation in treated cell lines, characterized by the presence of fragmented DNA bands at approximately 800bp along with a smear between 100bp and 200bp, strongly indicates extensive DNA damage associated with apoptosis. The appearance of distinct bands at 800bp suggests the cleavage of DNA into specific fragments, a typical feature of apoptotic cells undergoing programmed degradation of chromosomal DNA. Additionally, the presence of a smear pattern in the lower molecular weight range (between 100bp and 200bp) further confirms the occurrence of DNA fragmentation, indicative of the random cleavage of DNA into smaller fragments during the late stages of apoptosis. Similarly, plant-based products like fenugreek seeds have shown extensive fragmentation in MCF-7, HepG2 and HCT-116 cell lines; quercetin dihydrate, gallic acid, and naringin fragmented DNA in SiHa cells by upregulating p53 and p21 genes [25,26].

The results attained from the gene expression analysis showed a significant downregulation of EGFR when treated with naringenin-7-O-glucoside, hereby, suggesting a potential inhibitory effect. EGFR is a crucial proto-oncogene involved in numerous signalling pathway which plays a role in cell growth, proliferation, and differentiation. Hence, targeting, and inhibiting onco-EGFR will bring down cancers associated with various genes in the downstream pathway of EGFR, thereby, confirming the potential of the compound as an anticancer agent. A study by Hajrah et al. [26] demonstrated the effect of Ricinus extract on MCF7 cells leading to a downregulation of PIK3R3 oncogene DPP4 oncogene and the upregulation of PPAR-γ expression. Curcumin downregulates numerous oncogenes like c-Myc, N-Myc, cyclin-D1, Bcl-2, and Bcl-xL [27].

In numerous cancer studies, various other flavanone glycosides have also been shown to be anticancerous. Prunetionoside, a flavonoid compound, has potent cytotoxic effects and can cause necroptosis in AGS gastric cancer cells. Similarly, scutellarein, another flavonol, initiates an extrinsic pathway of apoptosis in HepG2 liver cancer cells and decreases the expression of cyclin protein [28]. In the optimization of therapy and in the search for new anticancer agents, the biological potential, bioavailability, cost-effectiveness, and minimal side effects of flavonoids make them promising cytotoxic anticancer agents.

The research presents promising findings; however, the study also has various limitations. The cell lines frequently lack the tumour microenvironment heterogeneity, thus, failing to know the impact of the drug on other factors including progression, angiogenesis and tumour-stromal interaction. The study does not provide information about the molecular mechanism involved in the downstream process of EGFR. In spite of the limitations, cell-based methods of cytotoxicity, fragmentation and DNA and gene expression analysis remain vital in the initial phases of anticancer drug development.

## Conclusions

To conclude, the results obtained in the study reveal the inhibiting potential of naringenin-7-O-glucoside against the aggressive TRBC form. With the apparent cytotoxic selectivity for the cancerous cells over the normal ones, the compound demonstrates the necessity of targetedness in anti-cancer therapies, aimed at reducing collateral damage associated with conventional chemotherapeutic interventions. Naringenin-7-O-glucoside exhibits apoptosis in TNBC cell lines as evidenced by the DNA fragmentation results. Moreover, the downregulation of EGFR expression highlights the potential of the compound to interfere with crucial signalling pathways involved in cancer progression. Exploiting the therapeutic potential of natural compounds like naringenin-7-O-glucoside represents a promising avenue for developing safer and more effective cancer treatments in the future.
